# 

**Published:** 1877-01

**Authors:** 


☞ We mail this (January) number to all last year's subscribers. No future numbers will be sent except to those who renew their subscriptions; and to avoid disappointment, renewals should be made at once.

VOL. XXXIV.
NUMBER 1.

January, 1877.

NEW SERIES,
VOL. 2.
NUMBER 1.

276

THE

### CHICAGO MEDICAL Journal and Examiner

PUBLISHED MONTHLY.

**EDITOR:**
WILLIAM H. BYFORD, A.M., M.D.

**ASSOCIATE EDITORS:**

JAMES H. ETHERIDGE, M.D. | JAS. NEVINS HYDE, A.M., M.D.

NORMAN BRIDGE, M.D. | FERD. C. HOTZ, M.D.

Published under the auspices of the

*CHICAGO MEDICAL PRESS ASSOCIATION.*

**TERMS—FOUR DOLLARS PER ANNUM, IN ADVANCE.**
**POSTAGE FREE.**

FOLIA SUA AD SANITATEM GENTIUM

CHICAGO:
W. B. KEEN, COOKE & CO., Publishers,
NOS. 113 AND 115 STATE STREET.

☞ Our Patrons and Contributors will kindly assist The Chicago [illegible]

